# Relationship between adipokines and androgens in children and young adults with congenital adrenal hyperplasia

**DOI:** 10.3389/fendo.2024.1433378

**Published:** 2024-08-08

**Authors:** Jennifer Apsan, Oksana Lekarev, Charlene Thomas, Yuan-Shan Zhu, Kaela Cohan, Karen Lin-Su

**Affiliations:** ^1^ Division of Pediatric Endocrinology, Department of Pediatrics, Weill Cornell Medicine, New York, NY, United States; ^2^ Division of Biostatistics, Department of Pediatrics, Weill Cornell Medicine, New York, NY, United States; ^3^ Clinical and Translational Science Center and Division of Endocrinology, Diabetes and Metabolism, Department of Medicine, Weill Cornell Medicine, New York, NY, United States

**Keywords:** CAH, adiponectin, visfatin, glucocorticoid, 21-hydroxylase deficiency

## Abstract

**Introduction:**

Children and young adults with congenital adrenal hyperplasia (CAH) are at increased risk of obesity and insulin resistance. There is evidence that children with CAH have increased visceral adiposity, which has been linked to metabolic syndrome and cardiovascular disease (CVD). The adipokine adiponectin has been shown to correlate with reduced metabolic risk, whereas the adipokines visfatin and leptin have been linked to visceral fat and adipocyte inflammation and can serve as biomarkers of increased metabolic risk. Few studies to date have characterized adipokine levels in children and young adults with congenital adrenal hyperplasia. We sought to investigate the relationship between adiponectin, leptin and visfatin levels to metabolic risk factors and androgen levels in children and young adults with CAH.

**Methods:**

Fasting blood was obtained for visfatin, leptin, adiponectin, glucose, insulin, CRP, lipid panel, total cholesterol (TC), triglycerides (TG) and HbA1c, as well as standard laboratory tests to assess adrenal control, from children with CAH due to 21-hydroxylase deficiency. HOMA-IR was calculated based on fasting glucose and insulin. Anthropomorphic measurements of BMI and waist-to-hip ratio were also obtained.

**Results:**

Adiponectin and androstenedione were inversely correlated (R = -0.57, p =0.016). There was a positive correlation between leptin and BMI percentile (R = 0.63, p <0.001) as well as leptin and HOMA-IR (R = 0.63, p <0.01). Glucocorticoid dose had a positive correlation with HOMA-IR (R=0.56, p = 0.021). Visfatin was inversely correlated with HDL cholesterol (R = -0.54, p = 0.026) and total cholesterol (R = -0.49, p <0.05). Overweight children and young adults had a significantly higher leptin (p = 0.02) and HOMA-IR (p=0.001) than non-overweight children and young adults.

**Conclusion:**

The inverse relationship between adiponectin and androstenedione suggests that better CAH control can reduce the risk of insulin resistance and metabolic syndrome. However, a high glucocorticoid dose appears to increase the risk of insulin resistance, underscoring the delicate balance required when treating CAH.

## Introduction

Children and young adults with congenital adrenal hyperplasia (CAH) are at increased risk for obesity and other components of metabolic syndrome, such as hypertension and insulin resistance (1–3). Previous studies also show evidence of increased abdominal fat as well as markers of obesity, inflammation, and insulin resistance that correlate with visceral fat (4, 5). Metabolic risk may be secondary to supraphysiologic glucocorticoid treatment or androgen excess, or both (1, 6).

Adiponectin, the most abundant type of adipokine secreted from adipocytes, is inversely related to adipocyte mass such that low levels occur in the setting of insulin resistance, type 2 diabetes, metabolic syndrome and cardiovascular disease (7, 8). Leptin is another well-known adipocyte derived adipokine that acts as a neural regulator of obesity, metabolic function and feelings of satiety. Visfatin, a newer and lesser known adipokine, is thought to reflect visceral fat and to mimic the effects of insulin-like stimulation of glucose uptake in adipocytes; more recently, it has been implicated in inflammation, endothelial dysfunction and atherosclerosis (9, 10).

A prior study reported adiponectin levels to correlate inversely with BMI, DHEAS and testosterone, but correlations to glucocorticoid dose in CAH and androstenedione were not described. The same study found elevated adiponectin levels in 51 children with CAH compared to BMI-matched controls, though the clinical relevance of this finding remains unknown (11). Another study reported that low adiponectin levels, along with adipocyte size, were the strongest predictors of insulin resistance in women with polycystic ovarian syndrome (PCOS), a syndrome related to androgen excess (12). Moreover, multiple studies have described lower levels of adiponectin in women with PCOS compared to BMI-matched controls (13). Leptin’s role in obesity and metabolic syndrome in women with PCOS is well-described (14), and one study also found elevated leptin levels in children with CAH (15). To our knowledge, there are no studies reporting visfatin levels in children and young adults with CAH. Visfatin levels have been found to be elevated in women with PCOS with higher levels in those with hyperandrogenemia compared to those without (10). There seems to be an important interplay between adipokine levels, androgen excess and metabolic markers, yet little is known about adipokine levels in children with CAH. Our study aimed to measure adiponectin, leptin, and visfatin levels in a small cohort of children and young adults with CAH aged 7 to 22 years and correlate them to glucocorticoid dose, BMI percentile, waist-to-hip ratio, total cholesterol, high-density lipoprotein cholesterol (HDL), low-density lipoprotein cholesterol (LDL), 17- hydroxyprogesterone (17OHP), androstenedione and testosterone.

## Methods

The study design was a prospective cross-sectional observational study. Subjects with a diagnosis of CAH due to 21-hydroxylase deficiency, ages 7–22 years, were recruited from the pediatric endocrinology clinic at New York Presbyterian/Weill Cornell Medical Center (NYP/WCMC). Those with metformin use, diabetes, chronic renal disease, chronic liver disease, heart failure, acute or chronic infections, or malignancies were excluded from the study. Written parental consent and assent from minors were obtained from participants in the pediatric endocrinology clinic. Adult patients signed a consent form and minor children signed an assent form with consent obtained from their caregivers.

At the study visit, fasting blood was collected for visfatin, leptin, adiponectin, plasma glucose (mg/dL), insulin (uIU/mL), CRP, lipid panel and HbA1c on patients who were already having routine laboratory tests drawn to assess adrenal hormone control (including 17OHP, androstenedione, and testosterone). Laboratory tests were obtained 1–3 hours after the morning hydrocortisone dose. HOMA-IR (a measure of insulin resistance) was calculated as [(fasting glucose (mg/dL) x insulin (uIU/mL)]/405. Total daily hydrocortisone dose (or hydrocortisone equivalent) was calculated as mg/m^2^/day. For subjects who were also receiving dexamethasone, a 40:1 conversion was used to calculate the hydrocortisone equivalent dose. We also obtained anthropomorphic measurements of waist-to-hip ratio and BMI. All participants received a $10 electronic gift card. This study was approved by the Weill Cornell Medicine Institutional Review Board.

The blood concentrations of insulin, plasma glucose, lipid panel and HbA1c were obtained from the hospital central laboratory. Assays for 17OHP, androstenedione, and testosterone were conducted on serum using chromatography/mass spectrometry. The serum concentrations of adiponectin, leptin, visfatin, and CRP were determined in the Core Laboratory of Clinical and Translational Science Center, Weill Cornell Medicine using ELISA kits from R&D Systems (Minneapolis, MN), Mediagnost (Reutlingen, Germany), IBL (Minneapolis, MN), and electrochemiluminescent kit from Meso Scale Diagnostics (Rockville, MD), respectively. The intraassay and interassay variations (CV%) are <6.5% and <6.9% for adiponectin, <8.7% and <14.0% for leptin, <6.3% and <7.2% for visfatin, <4.1% and <10.5% for CRP, respectively. The sensitivities of these tests are 0.0039 ug/mL for adiponectin, 1 ng/mL for leptin, 0.125 ng/mL for visfatin, and 0.0128 ng/mL for CRP.

### Statistical methods

Wilcoxon rank sum test (uses normal approximation to calculate the p-value) and Wilcoxon rank sum exact test were utilized to compare the clinical variables of interest across different grouping variables. Spearman’s rank correlation was used to investigate the relationship between continuous variables of interest (age, adiponectin, leptin, visfatin, CRP, 17OHP, androstenedione, and testosterone) and 1) glucocorticoid dose (mg/m^2^/day of hydrocortisone equivalent) and 2) the general inflammatory/metabolic profile items. All p-values are two-sided with statistical significance evaluated at the 0.05 alpha level. As this project was exploratory, p-values were not adjusted for multiple comparisons. All analyses were performed in R (4.0.5) for Mac.

## Results

**Figure 1:**
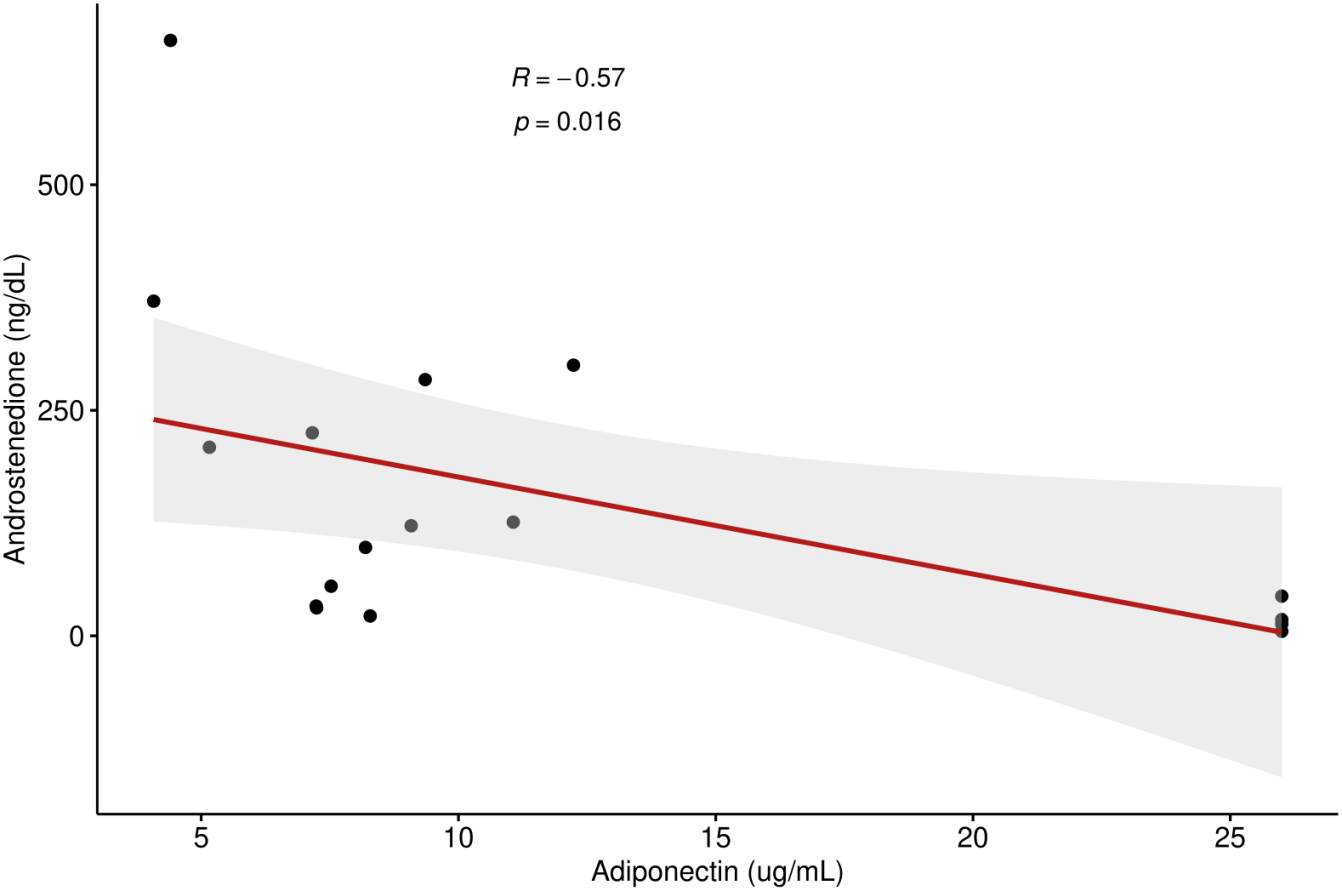
Correlation between androstenedione and adiponectin levels in children and young adults with CAH.

**Figure 2:**
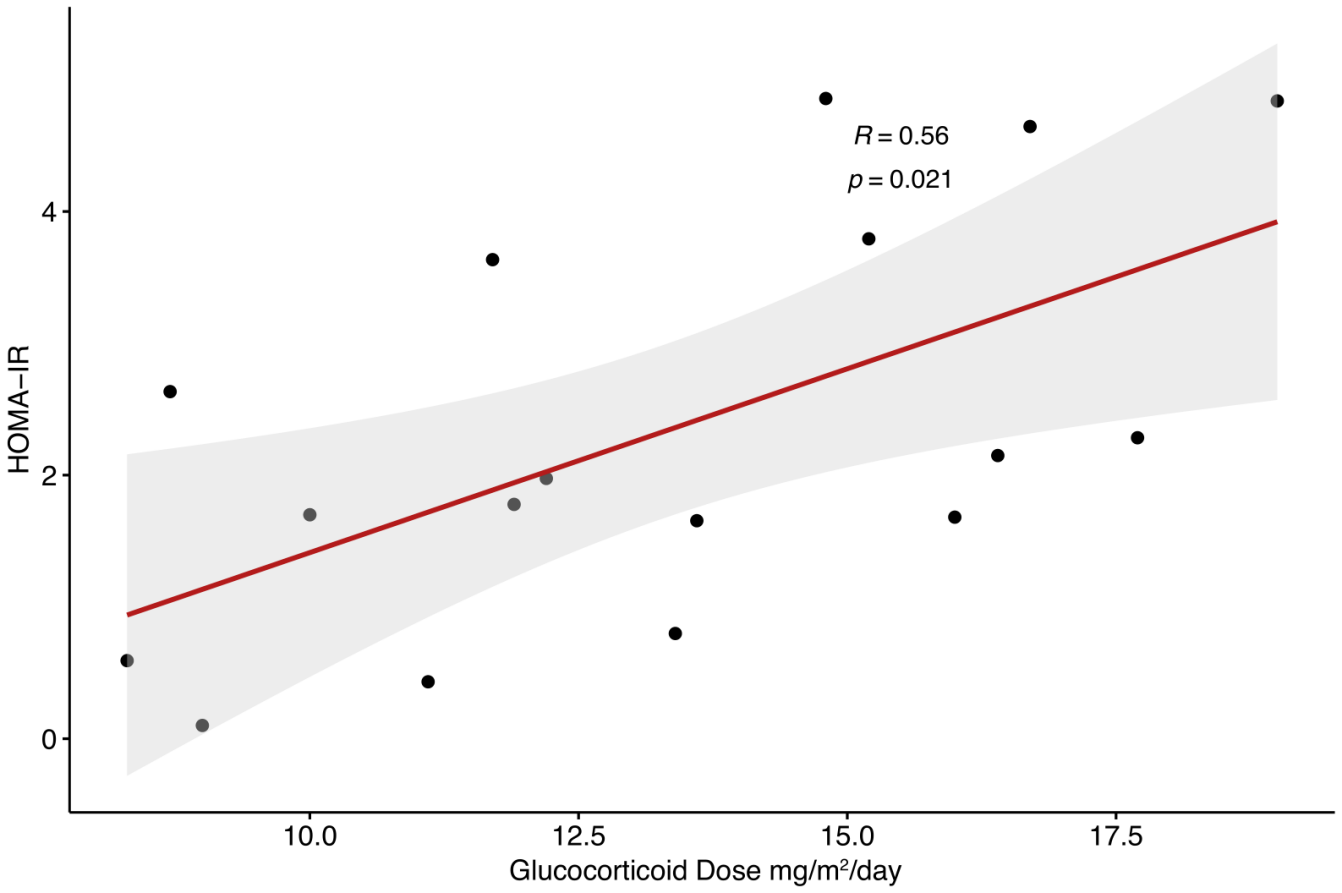
Correlation between glucocorticoid dose and HOMA IR in children and young adults with CAH.

A total of 17 children and young adults with CAH participated in this study (2M, 15F) (15 classical, 2 non-classical). Of the 15 classical CAH patients, 13 were salt-wasting (and taking fludrocortisone in addition to glucocorticoid) and 2 had simple-virilizing CAH. All participants had confirmed pathogenic variants in the CYP21A2 gene. Age range was 7–22 years of age. Results showed that adiponectin was inversely related to androstenedione (R = -0.57, p =0.016) (shown in **Figure 1**) but did not correlate to 17- hydroxyprogesterone or testosterone levels. Our results found a nonsignificant trend for correlation between adiponectin and HDL levels (R = 0.47, p= 0.058). No significant correlation was detected between adiponectin and BMI, waist-to-hip ratio, HOMA-IR, LDL, TG, or TC. Leptin, on the other hand, had a strong direct correlation to both HOMA-IR (R = 0.63, p <0.01) and BMI percentile (R = 0.63, p <0.001) but did not correlate with waist-to-hip ratio. Leptin had a nonsignificant trend for correlation to glucocorticoid dose (R = 0.47, p= 0.059) in children and young adults with CAH. Leptin levels did not correlate with TG, TC, HDL, LDL, 17OHP, androstenedione, or testosterone levels. Visfatin levels exhibited a strong inverse correlation to HDL (R = -0.54, p = 0.026) and total cholesterol (R = -0.49, p <0.05), but did not correlate with BMI, waist-to-hip ratio, HOMA-IR, LDL, TG, 17OHP, androstenedione, or testosterone in this population. Glucocorticoid dose had a significant positive correlation with HOMA-IR (R=0.56, p = 0.021) (**Figure 2**), but no correlation with adiponectin or visfatin.

Comparison between overweight and non-overweight subjects found significantly higher leptin and HOMA-IR levels, but significantly lower LDL and total cholesterol, in the overweight group (**Table 1**).

**Table 1:** Characteristics of non-overweight and overweight study participants.

Characteristic	Non Overweight, N = 11*^1^*	Overweight, N = 6*^1^*	p-value*^2^*
**Age**	10.3 (8.3, 12.9)	12.2 (10.0, 14.8)	0.7
**Glucocorticoid Dose (mg/m^2^/day of HC equivalent)**	11.90 (10.55, 13.50)	15.80 (14.90, 16.62)	0.10
**Adiponectin (ug/mL)**	8 (7, 19)	9 (7, 9)	0.8
**Leptin (ng/mL)**	6 (1, 9)	16 (13, 21)	0.020
**Visfatin (ng/mL)**	0.76 (0.42, 1.24)	1.54 (0.81, 1.91)	0.3
**CRP (ng/mL)**	842 (435, 10,406)	2,424 (1,127, 7,502)	0.6
**Systolic BP (mmHg)**	58 (34, 71)	72 (52, 94)	0.4
**Diastolic BP (mmHg)**	68 (56, 76)	82 (75, 93)	0.13
**W/H Ratio**	0.86 (0.84, 0.93)	0.90 (0.90, 0.94)	0.2
**HbA1c (%)**	5.10 (4.95, 5.35)	5.45 (5.25, 5.57)	0.086
**HOMA IR**	1.68 (0.70, 1.88)	4.22 (2.92, 4.79)	0.001
**Total Cholesterol (mg/dL)**	175 (166, 194)	136 (112, 149)	0.037
**Triglycerides (mg/dL)**	61 (52, 95)	58 (48, 68)	0.6
**HDL (mg/dL)**	68 (58, 70)	54 (40, 63)	0.2
**LDL (mg/dL)**	101 (94, 110)	64 (56, 82)	0.030

^1^Median (IQR).

^2^Wilcoxon rank sum exact test; Wilcoxon rank sum test.

## Discussion

Adipokines comprise a group of cytokines that are secreted mainly by adipose tissue and other tissue, such as placenta, ovaries, liver, heart and bone marrow, and are thought to play a significant role in metabolic dysfunction and general inflammation. They are known to have an effect on insulin sensitivity, inflammation, growth and development (16). Adiponectin, leptin and visfatin are adipokines that are arising as potential markers of visceral fat and insulin resistance (9, 17).

Our results found that adiponectin is inversely related to androstenedione (**Figure 1**) in our cohort of children and young adults with CAH, which is consistent with prior studies. In 2016, a study showed that administration of recombinant human adiponectin remarkably decreased ovarian androstenedione levels (18). Korner et al. confirmed a strong relationship of adiponectin to testosterone and DHEAS (19). Androgens of pubertal boys have also been shown to have a suppressive effect on adiponectin levels (20). This relationship was looked at specifically in children with CAH in a study by Völkl et al. (2009), which showed that serum testosterone and DHEAS levels negatively correlated with adiponectin levels in children with classical CAH (11). It is also consistent with prior studies showing lower adiponectin both in women with PCOS and adolescent boys with positive correlation to their increasing androgen levels, thus supporting a presumed hypothesis that insulin resistance is impacted by increasing androgen levels (3, 6). Androstenedione is an important adrenal androgen in CAH and its inverse correlation with adiponectin in CAH patients has not been reported to our knowledge but is consistent with the relationship to other known androgens. As low adiponectin appears to be the strongest predictor of metabolic syndrome in children and young adults, the correlation to elevated androstenedione, but not BMI or waist-to-hip ratio or glucocorticoid dose, may support elevated androgens as a stronger factor of metabolic risk than glucocorticoid treatment or obesity itself.

We found a nonsignificant trend for a direct relationship between adiponectin and HDL, which is consistent with previous studies looking at the interplay between adiponectin and HDL (21). Cholesterol efflux capacity (CEC) is the initial step in the pathway by which excess cholesterol is exported and packaged as HDL. Low adiponectin has been found in prior studies to be a robust predictor of poor CEC and low HDL in adults irrespective of BMI (22, 23). The lack of significance is most likely due to a small sample size, but this finding could have future implications for adiponectin as a biomarker of lipid health in children and young adults with CAH.

Leptin, an adipokine secreted from white adipose tissue, is implicated in modulation of energy homeostasis. It is secreted by adipocytes and signifies the amount of energy reserves stored in subcutaneous and visceral adipose tissue deposits. Both insulin and glucocorticoids have been shown to increase leptin levels while androgens can suppress levels (15). A prior study of 11 children with CAH showed an increase in leptin levels after initiation of hydrocortisone therapy (24). Leptin levels have also been shown to overall be higher in children with CAH even after correction for BMI. Mechanistically, this may be tied to low epinephrine and metanephrine, which serve to suppress leptin levels (15). Our results found that leptin levels correlate strongly with both HOMA-IR and BMI in children and young adults with CAH, serving to add to the understanding of propensity of these children and young adults to metabolic dysfunction. Interestingly, we did not find a correlation between androgen levels and leptin, which may be due to our small sample size. Leptin, unlike other studied adipokines, trended in a correlation to glucocorticoid dose, perhaps linking metabolic risk and glucocorticoid intake in children and young adults with CAH.

Visfatin levels were noted to correlate inversely to HDL levels, a finding that has also been identified in women with PCOS (10). We also found a negative correlation between visfatin levels and total cholesterol. This is not fully understood as prior studies have correlated visfatin and total cholesterol via a direct correlation (25). In our study, visfatin did not correlate with BMI, HOMA-IR or androgens. Further studies are needed to see if visfatin is a useful biomarker to understand metabolic risk in children and young adults with CAH.

There is a well-known link between supraphysiologic glucocorticoid dosing and insulin resistance, though some studies have noted increased HOMA-IR in CAH independent of glucocorticoid dose (26, 27). Our study, in contrast, found a significant correlation between HOMA-IR and glucocorticoid dose. One possible explanation is that patients with insulin resistance require higher doses of glucocorticoid in order to adequately suppress adrenal androgens. However, it may also indicate that a higher glucocorticoid dose is an independent risk factor for insulin resistance in patients with CAH on glucocorticoid therapy, which underscores the delicate balance required when treating individuals with CAH.

It was not surprising that overweight population had significantly higher leptin and HOMA-IR. LDL, however, was found to be lower in the overweight group. It should be noted that neither group’s LDL is considered high. In any event, this finding may underscore the multifactorial nature of hypercholesteremia, including nutritional impacts and genetic factors.

Our study was limited by a small sample size of 17 patients both because of constraints on funding the laboratory assay and the timing of the study in 2020–2021 during the beginning of the COVID-19 pandemic. Our intention, therefore, was for this study to serve as a foundational pilot study for further studies.

## Conclusion

Adipokines are secreted from adipose tissue and can serve as biomarkers of metabolic dysfunction. The extent of metabolic dysregulation that occurs in children and young adults with CAH is not fully understood and adipokine levels are not well established in this population. Our results found that adiponectin inversely correlates to androstenedione levels in children and young adults with CAH, adding to the understanding that hyperandrogenemia may exacerbate insulin resistance and metabolic dysfunction in children with CAH. Leptin, on the other hand, correlated strongly with BMI, HOMA-IR and trended towards a significant correlation with glucocorticoid dose, which suggests that metabolic risk in children with CAH is tied to supraphysiologic glucocorticoid dosing. Given the association between insulin resistance and glucocorticoid dose, treatment should be titrated to the lowest possible dose that still adequately controls hyperandrogenemia. More studies are required to see if visfatin can serve as an additional marker in understanding metabolic risk in children with CAH.

## Data availability statement

The raw data supporting the conclusions of this article will be made available by the authors, without undue reservation.

## Ethics statement

The studies involving humans were approved by Weill Cornell Medicine Institutional Review Board. The studies were conducted in accordance with the local legislation and institutional requirements. Written informed consent for participation in this study was provided by the participants’ legal guardians/next of kin.

## Author contributions

JA: Writing – original draft, Writing – review & editing, Conceptualization, Investigation, Methodology. OL: Writing – original draft, Writing – review & editing, Conceptualization, Data curation, Investigation, Supervision. CT: Writing – original draft, Writing – review & editing, Formal analysis. Y-SZ: Writing – original draft, Writing – review & editing, Methodology. KC: Writing – original draft, Writing – review & editing, Data curation. KL-S: Writing – original draft, Writing – review & editing, Data curation, Conceptualization, Investigation, Methodology, Supervision.
